# Accelerating the Design of High-Energy-Density Hydrocarbon Fuels by Learning from the Data

**DOI:** 10.3390/molecules28217361

**Published:** 2023-10-31

**Authors:** Linyuan Wen, Shiqun Shan, Weipeng Lai, Jinwen Shi, Mingtao Li, Yingzhe Liu, Maochang Liu, Zhaohui Zhou

**Affiliations:** 1 State Key Laboratory of Fluorine & Nitrogen Chemicals, Xi’an Modern Chemistry Research Institute, Xi’an 710065, China; 2 Xi’an Key Laboratory of Liquid Crystal and Organic Photovoltaic Materials, Xi’an 710065, China; 3 International Research Center for Renewable Energy, State Key Laboratory of Multiphase Flow in Power Engineering, Xi’an Jiaotong University, Xi’an 710049, China; jinwen_shi@mail.xjtu.edu.cn (J.S.);; 4 Xi’an Aerospace Propulsion Test Technique Institute, Xi’an 710064, China; 5 Department of Chemical Engineering, School of Water and Environment, Chang’an University, Xi’an 710064, China

**Keywords:** high-energy density, hydrocarbon fuels, high-throughput screening, density functional theory, materials design

## Abstract

In the ZINC20 database, with the aid of maximum substructure searches, common substructures were obtained from molecules with high-strain-energy and combustion heat values, and further provided domain knowledge on how to design high-energy-density hydrocarbon (HEDH) fuels. Notably, quadricyclane and syntin could be topologically assembled through these substructures, and the corresponding assembled schemes guided the design of 20 fuel molecules (ZD-1 to ZD-20). The fuel properties of the molecules were evaluated by using group-contribution methods and density functional theory (DFT) calculations, where ZD-6 stood out due to the high volumetric net heat of combustion, high specific impulse, low melting point, and acceptable flash point. Based on the neural network model for evaluating the synthetic complexity (SCScore), the estimated value of ZD-6 was close to that of syntin, indicating that the synthetic complexity of ZD-6 was comparable to that of syntin. This work not only provides ZD-6 as a potential HEDH fuel, but also illustrates the superiority of learning design strategies from the data in increasing the understanding of structure and performance relationships and accelerating the development of novel HEDH fuels.

## 1. Introduction

As the key component in engines, high-energy-density hydrocarbon (HEDH) fuels have always been the subjects of scientific concern [1,2], among which many qualified fuels stand out, such as exo-tetrahydrodicyclopentadiene (JP-10), quadricyclane (QC), and so on [3,4,5]. In pursuit of higher payloads, HEDH fuels must meet the required properties, such as high density, low melting point, high flashing point, high specific impulse [6]. To this end, successive researchers have devoted themselves to this field and are committed to designing and synthesizing new HEDH fuels [7,8,9], where cyclic hydrocarbons with a high stain energy usually possess a high density and high gravimetric net heat of combustion [10,11]. Compared to the traditional trial-and-error approaches, computational modeling and design has been established as an important way to accelerate the discovery of new materials with the rapid growth of computing power, by using high-throughput computations, machine learning, and quantum chemistry calculations [12,13,14,15,16,17,18,19,20,21]. Moreover, Xiao’s groups investigated the pentacyclo[5.4.0.0^2,6^.0^3,10^.0^5,9^]undecane (PCU) and its hydrocarbon derivatives, and expressed its promising application as an HEDH fuel through DFT studies [22]. Namboothiri’s group designed 1,4-disubstituted cubane derivatives and synthesized the desired molecules [23]. Moreover, Parakhin constructed a series of N,N′-methylene-bridged polynitro hexaazaisowurtzitane molecules and found the specific impulse higher than CL-20 [24]. Recently, Li and co-workers proposed an effective machine learning (ML) method enabling the high-throughput screening of next-generation fuels from the hydrocarbon subset of GDB-13 and further continued the idea with more advanced methods [25,26,27]. These approaches furnished the ways for designing novel fuels and offered some high-performance candidates. However, considering the “black box” of the ML models, it may lack some inherent information for the current fuels by collapsing multiple matrices. Therefore, how to grasp the characteristics of the existing fuels and provide further guidance for the rational design of new HEDH fuels is particularly important and attractive.

In recent years, the amount of data in various industries has grown exponentially; therefore, many researchers collected these data and built specialized databases, for example, ZINC20 [28] and Materials Project [29,30]. On the basis of these data, plenty of groups applied data mining approaches to exact the relation between performance and the specific structural fingerprint, elemental composition, and learned design rules for polymer dielectrics [31], transparent conducting materials [32], and so on [33]. These works inspired us and offered an alternative method of designing novel HEDH fuels through learning from the data.

**Figure 1:**
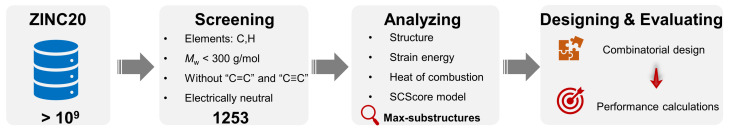
Illustration of the whole workflow.

Herein, as depicted in Figure 1, high-throughput screening is employed to obtain the molecules with potential as HEDH fuels from the ZINC20 database, and the structural features are mined by a statistical analysis as well as the maximum substructure search approach to improve the domain knowledge. More importantly, the topologically assembled schemes of syntin and QC using the common substructures of the above-screened candidates are excavated and combined with the combinatorial design strategy to further guide the design of 20 fuels. Ultimately, these designed fuels are evaluated with group-contribution methods and DFT calculations, all exhibiting higher volumetric net heat values of combustion than QC, illustrating the potential applications for HEDH fuels and the effectiveness of the proposed approach.

## 2. Results and Discussion

**Figure 2:**
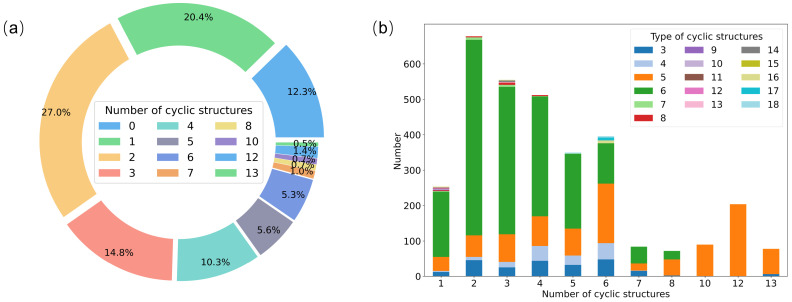
(**a**) Distribution map of the types of molecules with different numbers of cyclic structures after screening; (**b**) distribution map of type of cyclic structures in different types of molecules after screening.

Since strained cyclic hydrocarbons release more energy compared to chain-structured fuels [34,35,36], more attention was given to features of cyclic hydrocarbons in the current work. Figure 2a shows the statistical distribution of molecule types presenting different numbers of cyclic structures, where only 12.3% of the molecules lack cyclic structures, while most of the molecules (over 60%) have 1~3 cyclic structures. Meanwhile, the proportion of corresponding molecule types decreases sharply, with the number of cyclic structures in the molecules increasing. This may be related to the increased difficulty of synthesis caused by the increase in the number of intermolecular cyclic structures, which is further manifested in a much lower number of corresponding molecules with more cyclic structures. Having analyzed the distribution of molecule types with different numbers of cyclic structures, our attention was turned to figuring out the distribution of different types of cyclic structures in different molecule types. It can be seen from Figure 2b that three-, four-, five-, and six-membered cyclic structures account for an overwhelming majority in the entire cyclic structure, which can be ascribed to the relatively easier synthesis. Moreover, the six-membered cyclic structures are dominant when the number of cyclic structures less than eight, while the five-membered structures hold the major proportion when the number of cyclic structures is higher than eight.

**Figure 3:**
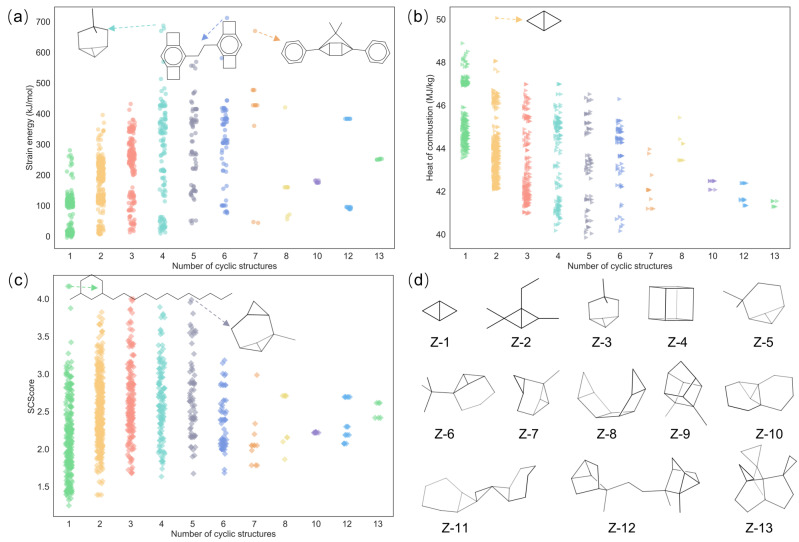
(**a**) Strain energy distribution, (**b**) heat of combustion distribution, (**c**) SCScore distribution among molecules containing different numbers of cyclic structures; (**d**) structures of selected molecules. The colors are consistent with the molecular classes in Figure 2a.

Considering the abovementioned structural analysis and the role of cyclic structures in fuel molecules [37], the strain energy, heat of combustion, and SCScore were all quantified for the cyclic molecules, and their respective distributions in molecules containing different numbers of cyclic structures are illustrated in Figure 3. As can be seen in Figure 3a, the strain energy shows an upward trend as the number of ring structures in the molecule increases to six. Considering the inherent high strain energy of three- and four-membered cyclic structures (29.0 and 26.3 kcal/mol) [38,39], this trend can be explained from the observation in Figure 2b. In detail, the proportion of strained three- and four-membered cyclic structures among the corresponding molecule types increases until the molecule contains six cyclic structures. Specifically, the molecules with relatively high strain energies actually hold the strained three- or four-membered cyclic structures, as shown in Figure 3a, which is in good agreement with the abovementioned analysis. Figure 3b illustrates the heat of combustion distribution among the molecules containing different numbers of cyclic structures. It was found that the heat of combustion decreased with the increasing number of cyclic structures in the molecules. This was because the gain in the enthalpy of formation from the increase in the number of cyclic structures was not as great as the increase in the molar mass in the denominator term (see Equation (S14) in Supplementary Materials). Bicyclo[1.1.0]butane possessed the highest heat of combustion value, showing the superiority of the fused two three-membered cyclic structure in improving the heat of combustion and providing an effective way to design high-heat-of-combustion fuels [37]. Then, the SCScore distribution among the molecules containing different numbers of cyclic structures is illustrated in Figure 3c. It is worthwhile mentioning that the synthetic complexity of the molecule was related to the number of cyclic structures in itself, roughly showing the rule that the lower the number of cyclic structures in the molecules, the easier it is to be synthesized. Additionally, the two molecules shown at the top of Figure 3c also illustrate that the long-chain and stereoscopic structures lead to a relatively higher synthetic complexity. In order to further learn the guidance for HEDH fuel designs, 13 molecules with a strain energy over 300 kJ/mol and heat of combustion value over 45 MJ/kg were selected, as shown in Figure 3d, and the corresponding values are listed in Table S1. These molecules possessed almost fused cyclic or caged structures, which were proposed as an effective way to improve the energy density of fuels [37].

**Figure 4:**
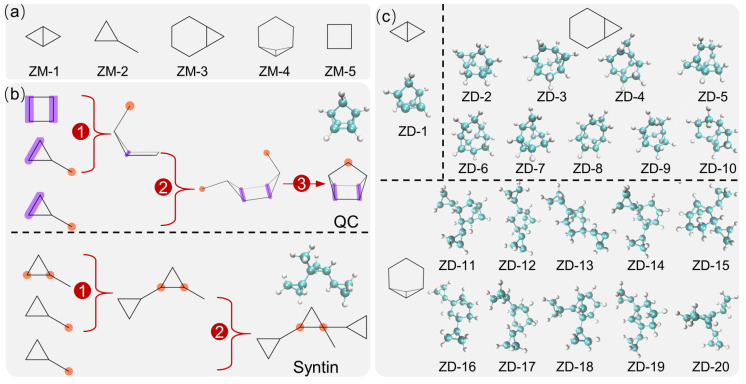
(**a**) Demonstration for the max-substructures of the selected molecules; (**b**) diagram of the two assembled schemes for QC and syntin using the max-substructures, where purple bonds represent the sharing bond and orange circles represent the bridging positions for generating structures; and the 1–3 represent the steps of assembly; (**c**) illustration of designed fuel molecules.

Among the selected molecules, the maximum common substructure search approach was applied to obtain the structural features, and the top-five most frequent max-substructures of these molecules (labeled ZM-1 to ZM-5) are depicted in Figure 4a. They almost have strained three- or four-membered cyclic structures, as well as fused structures, indicating their advantages in building HEDH fuels. Figure 4b shows the different structural topological assembled schemes for QC and syntin fuels. It was found that the QC could be topologically assembled from one ZM-5 and two ZM-2 structures by sharing bond and bridging processes. In detail, the intermediate structure was constructed with two ZM-2 structures fused with ZM-5 (by sharing bonds colored in purple), which finally bridged the orange sites to form a caged QC topologically. Additionally, syntin could be generated from three ZM-2 structures; specifically, the two ZM-2 structures in turn bridged the orange sites of the initial ZM-2 structure. These two assembled schemes learned from the topological structures provided subsequent design rules. It was noteworthy that the QC-assembled scheme formed the caged structures, probably leading to an increase in the synthetic complexity. Since ZM-4 possessed a relatively complex stereoscopic structure, considering the synthetic complexity, the syntin-assembled scheme was employed for ZM-4, while the QC-assembled scheme was applied to two other max-substructures (ZM-1 and ZM-3). Since QC was constructed using a combination of ZM-5 and two ZM-2 structures, and applying the syntin-assembly scheme to ZM-5 would have increased the difficulty of synthesis, ZM-5 was not considered in the subsequent design. Through removing duplicate structures, 20 designed fuels (labeled ZD-1 to ZD-20) from the proposed assembled schemes are shown in Figure 4c.

**Figure 5:**
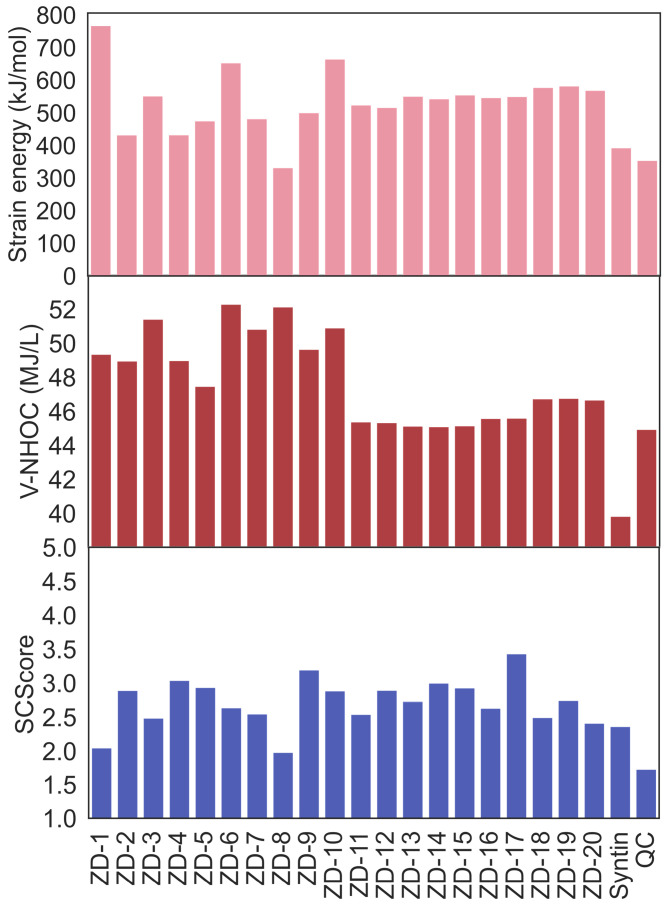
Diagram of strain energy, volumetric net heat of combustion, and SCScore of designed fuels.

Furthermore, Figure 5 manifests the value of the strain energy, volumetric net heat of combustion (V-NHOC), and SCScore of the designed fuels after the calculations of the group-contribution methods and DFT. The great majority of the designed fuels possessed higher strain energies than the QC and syntin, which could be ascribed to the more strained three-membered cyclic and fused stereoscopic structures in the molecules. It is worth mentioning that ZD-1 was constructed by ZM-1 and two ZM-2 structured through the QC-assembled scheme. In other words, ZD-1 had a cage-like structure fused by the four three-membered cyclic structures, thus endowing it with a strain energy exceeding 700 kJ/mol. Moreover, Table 1 lists the estimated properties after the DFT calculations and Glushko’s formula, including the density, net heat of combustion (NHOC), melting point (*T*_m_), flash point (*FP*), and *I*_sp_. The designed fuels from ZD-1 to ZD-10 with the QC-assembled scheme had a higher density than the other fuels generated with the syntin-assembled scheme, which could be attributed to the additional fused structures in the structure generation [37]. Meanwhile, since V-NHOC was equal to the density multiplied by the NHOC, the higher density and NHOC endowed a decent V-NHOC value, as shown in Figure 5, where the V-NHOCs of the design fuels were higher than that of the QC, showing the effectiveness of the design strategy for HEDH fuels. Considering the application in liquid fuels, the designed fuels (including ZD-8 and ZD-11 to ZD-17) with *T*_m_ higher than 273.15 K were not suitable [25]. Moreover, the *FP* value of designed fuels representing the lowest temperature at which the vapor of fuels ignites while in contact with a flame would be better at temperatures higher than 38 °C [40], and several designed fuels met this requirement. Further taking the values of specific impulses and synthetic complexities into account, ZD-6 stood out due to the *I*_sp_ with a value of 358.5 s and an acceptable SCScore value of 2.64, showing the potential for its application as an HEDH fuel.

## 3. Conclusions

In this work, high-throughput screening was applied to search the ZINC20 database for molecules with the potential to be used as HEDH fuels. Among the screened 1253 molecules, the structure and performance distribution among various types and numbers of ring structures in the molecule were statistically analyzed, and five common substructures were obtained with the aid of max-substructure search methods for the subsequent design. In addition, the topologically assembled schemes of QC and syntin by the common substructures were learned from these data, and thus used to guide the design of 20 fuels. Among them, ZD-6 had an excellent performance, with a V-NHOC of 52.3 MJ/L and an *I*_sp_ of 358.5 s, as evaluated by the group-contribution methods and DFT calculations. Furthermore, according to the neural network model for synthetic complexity, ZD-6 was predicted to have an SCScore value close to that of syntin, showing a comparable synthesis complexity to syntin. All of these not only reflect the superiority of ZD-6 in potential applications, but also validate the effectiveness of the proposed strategy learned from the data in designing HEDH fuels. This study will expand our understanding of the design of HEDH fuels regarding multiple properties and open new avenues for the design of organic functional materials.

## Figures and Tables

**Table 1:** Estimated properties of designed fuels as well as syntin and QC.

Index	*ρ* (g/cm^3^)	NHOC (MJ/kg)	*T*_m_ (K)	*FP* (K)	*I*_sp_ (s)
ZD-1	1.03	47.94	127.4	229.4	371.6
ZD-2	1.11	44.10	249.0	308.0	350.8
ZD-3	1.14	45.27	265.9	318.6	355.5
ZD-4	1.10	44.40	267.8	319.6	352.0
ZD-5	1.07	44.44	251.1	309.6	352.2
ZD-6	1.14	46.05	265.9	318.6	358.5
ZD-7	1.14	44.75	265.9	318.6	353.4
ZD-8	1.19	43.89	281.0	327.9	350.0
ZD-9	1.11	44.86	229.4	317.1	353.9
ZD-10	1.11	45.86	249.0	308.0	357.8
ZD-11	1.01	44.96	310.2	376.0	351.5
ZD-12	1.01	44.92	310.2	376.0	351.3
ZD-13	1.01	44.89	273.9	366.9	351.2
ZD-14	1.01	44.85	273.9	366.9	351.0
ZD-15	1.01	44.91	273.9	366.9	351.3
ZD-16	1.02	44.89	288.5	366.8	351.2
ZD-17	1.02	44.90	288.5	366.8	351.2
ZD-18	1.04	44.85	263.0	356.3	351.0
ZD-19	1.04	44.88	263.0	356.3	351.1
ZD-20	1.04	44.80	263.0	356.3	350.8
syntin	0.88	45.34	222.9	324.1	349.8
QC	1.00	45.01	230.0	273.7	355.4

## Data Availability

The data are available from the corresponding authors upon request.

## Supplementary Materials

The following supporting information can be downloaded at: https://www.mdpi.com/article/10.3390/molecules28217361/s1. The Materials and Methods Section shows the specific calculation details. Figure S1 and the corresponding description show the details of the strain energy calculation. Table S1 lists the NHOC, strain energy, and SCScore of 13 selected molecules. Table S2 and Equations (S1)–(S16) demonstrate how to calculate the fuel properties using group-contribution methods and DFT calculations. Table S3 lists the XYZ coordinates of the designed fuel molecules. Table S4 lists the electronic and thermal corrections to the energies, enthalpies, and free energies of the designed fuel molecules. Table S5 lists differences in strain energy values between the PM7 and DFT methods for designing the fuel molecules. Refs. [6,25,40,41,42,43,44,45,46,47,48] are cited in Supplementary Materials fie.
