# Supplementary material for: Accelerating the Design of High-Energy-Density Hydrocarbon Fuels by Learning from the Data
Source: Molecules. 2023 Oct 31;28(21):7361. doi: 10.3390/molecules28217361 (PMC10647593; doi:10.3390/molecules28217361)
Supplement: Supplementary file 1 [file molecules-28-07361-s001.zip › molecules-2676465-supplementary.pdf]

Supporting Information

Accelerating the Design of High-Energy-Density Hydrocarbon Fuels by Learning from the Data

Linyuan Wen [1,2,†], Shiqun Shan [3,†], Weipeng Lai [1], Jinwen Shi [4], Mingtao Li [4], Yingzhe Liu [1,2,*], Maochang Liu [4] and Zhaohui Zhou [5,*]

[1] State Key Laboratory of Fluorine & Nitrogen Chemicals, Xi'an Modern Chemistry Research Institute, Xi'an 710065, China; wenlinyuan_204@163.com (L.W.); lwpdry@126.com (W.L.)
[2] Xi'an Key Laboratory of Liquid Crystal and Organic Photovoltaic Materials, Xi'an 710065, China
[3] Xi'an Aerospace Propulsion Test Technique Institute, Xi'an 710064, China; ssq3828@163.com
[4] International Research Center for Renewable Energy, State Key Laboratory of Multiphase Flow in Power Engineering, Xi'an Jiaotong University, Xi'an 710049, China; jinwen_shi@mail.xjtu.edu.cn (J.S.); mingtao@mail.xjtu.edu.cn (M.L.); maochangliu@mail.xjtu.edu.cn (M.L.)
[5] Department of Chemical Engineering, School of Water and Environment, Chang'an University, Xi'an 710064, China
* Correspondence: liuyz_204@163.com (Y.L.); zzhlax@chd.edu.cn (Z.Z.)
† These authors contributed equally to this work.

1. Methods

The ZINC20 database was considered in the current investigation due to its large amount of molecule data exceeding 10^9. To screen the molecules with suitable fuel properties in the database, high-throughput screening with the aid of the RDKit package [1] included four aspects: elements, molecular weight, substructures, and electric neutrality. Since this contribution focused on designing HEDH fuels, the molecules containing elements other than carbon and hydrogen were filtered out and were electrically neutral. Considering the molecular weight of the current quantified fuels as well as the computational costs, the molecular weight threshold was set to 300 g/mol. At the same time, since olefinic and acetylenic bonds had the ability to store more hydrogen; in other words, the molecules that contained these two types of bonds did not perform well owing to the inefficient energy storage properties. Thus, the molecules containing these two specific bond types were not considered in the current work.

Subsequent to performing the abovementioned high-throughput screening, 1253 molecules were screened out and analyzed in terms of the structure distribution, strain energy, heat of combustion, and synthetic complexity, to furnish the domain knowledge for guiding subsequent HEDH fuel designs. For the trade offing calculations of the speed and accuracy, the PM7 method [2] was used in this part for the evaluation of the strain energy from homodesmotic reactions [3-4] and heat of combustion. The SCScore employed a neural network model for predicting the synthetic complexity with the expected number of reaction steps (from 1 to 5, with higher values representing a greater synthesis complexity) and was adopted in the current work to filter difficult-to-synthesize molecules[5]. In the quest for structural commonalities between favorable molecules with a high performance value, the max-substructures were selected through the maximum common substructure search approach [1].

It was found that syntin and QC could be topologically assembled from the max-substructures, as previously mentioned, and 20 novel fuels were designed from the remaining max-substructures according to

the learned assembled schemes as well as the combinatorial design strategy. According to the previous reports, the group-contribution approach [6-9] was adopted for predicting the physicochemical and energy properties of the designed HEDH fuels, in cooperation with the DFT simulations at a B3LYP/6-311G** level by Gaussian 16 [10] for calculating the electronic and thermal correction energies, enthalpies, and free energies, as well as the strain energy. The specific impulse (I_{sp}) of the designed fuel was evaluated by an empirical equation, as shown in the Supplementary Materials [8, 11].

2. Strain energy calculations regarding the homodesmotic reaction.

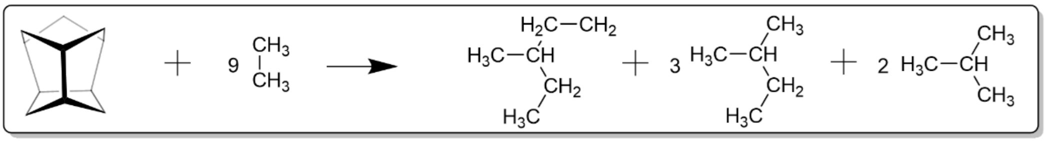

Figure S1. Illustration of the homodesmotic reactions as an example.

The strain energies are evaluated using the following formula: $E_s = \sum_R(n_iE_i + n_iZPE_i) - \sum_P(n_jE_j + n_jZPE_j)$, where E_s, E_i, E_j represent the strain energies, total energy of the ith reactant, and total energy of the jth product, respectively; n_i and n_j represent the the stoichiometric numbers in the homodesmotic reactions; and ZPE_i and ZPE_j represent the zero-energy values of the ith reactant and j^{th} product, respectively.

3. Net heat of combustion (NHOC), strain energy, and SCScore values of the 13 selected fuels calculated at the PM7 level.

Table S1. NHOC, strain energy, and SCScore values of the 13 selected fuels.

Name	NHOC (MJ/kg)	Strain Energy (kJ/mol)	SCScore
Z-1	50.06	328.96	1.56
Z-2	48.04	314.75	2.05
Z-3	46.98	335.52	2.81
Z-4	46.29	580.96	1.73

Z-5	46.98	335.54	2.81
Z-6	46.98	341.25	2.58
Z-7	46.52	439.71	2.39
Z-8	46.26	556.31	2.03
Z-9	45.29	418.05	2.07
Z-10	45.94	338.82	2.81
Z-11	45.61	378.33	2.18
Z-12	45.43	420.38	2.15
Z-13	45.03	383.78	2.17

4. Group-contribution methods and DFT calculations.

Table S2. Groups and corresponding contributions for estimating the properties (G_i)[7].

Group	T_m	T_b	T_c	P_c	V_c	$\Delta_{vap}H$
CH3	0.6953	0.8491	1.7506	0.018615	68.35	0.217
CH2	0.2515	0.7141	1.3327	0.013547	56.28	4.910
CH	-0.3730	0.2925	0.596	0.007295	37.5	7.962
C	0.0256	0.0386	-0.2399	0.003268	33.32	7.017
(CH3)2CH	0.1175	-0.0035	-0.0471	0.000473	1.71	-0.399
(CH3)3CH	-0.1214	0.0072	-0.1778	0.00034	3.14	-0.417
CH_{cycle}-CH2	-0.4669	-0.0148	0.3816	0.001694	-2.95	-0.428
CH_{cycle}-CH	-0.3548	0.1395	0.1093	0.000124	6.19	0.153
CH_{cycle}-C	-0.1727	0.1829	0.0	0.0	0.0	0.0

C_{cycle}-CH2	-1.9233	0.0319	0.109	-0.00061	-5.17	0.585
$CH2_{cycle}$	0.5699	0.8234	1.8815	0.009884	49.24	3.341
CH_{cycle}	0.0335	0.5946	1.102	0.007596	44.95	6.416
C_{cycle}	0.1695	0.0386	-0.2399	0.003268	33.32	7.017
CH_{cycle}-CH3	-0.1326	-0.1210	-0.1233	0.000779	2.79	0.096
C_{cycle}-CH3	0.1737	0.0722	0.1607	0.001235	1.95	0.808
CH_{cycle}-CH_{cycle} (Different rings)	0.5460	0.4387	2.1761	0.002745	7.72	-2.425
$CH_{multicycles}$	0.6647	0.1415	1.2513	-0.0045	-3.33	-2.095
$C_{multicycles}$	0.0792	0.2	1.3	0.0	0.0	-2.7

n_i represents the number of group-types *i* in **Table S2**.

Melting point (T_m/K):

$$\exp\left(\frac{T_m}{147.45}\right) = \sum_i n_i G_i \tag{S1}$$

Normal boiling point (T_b/K):

$$\exp\left(\frac{T_b}{222.543}\right) = \sum_i n_i G_i \tag{S2}$$

Critical temperature (T_c/K):

$$\exp\left(\frac{T_c}{231.239}\right) = \sum_i n_i G_i \tag{S3}$$

Critical pressure (P_c/bar):

$$(P_c - 5.9827)^{-0.5} - 1.08998 = \sum_i n_i G_i \tag{S4}$$

Critical volume (V_c/cm^3•mol^{-1}):

$$V_c - 7.95 = \sum_i n_i G_i \tag{S5}$$

Standard enthalpy of vaporization at 298 K ($\Delta_{vap}H$/ kJ•mol^{-1}):

$$\Delta_{vap}H - 11.733 = \sum_i n_i G_i \tag{S6}$$

The flash point (*FP*/K) at an atmospheric pressure can be calculated from Equation (S7):

$$FP = 1.477 \times T_b^{0.8} \times \Delta_{vap}H^{0.17} \times n^{-0.06} \quad \text{(S7)}$$

where n represents the number of carbon atoms in the fuel molecule.

Density at 298 K can be calculated from the following equations. M is the molar mass (g/mol):

$$\omega = \frac{3}{7} \cdot \frac{\frac{T_b}{T_c}}{1-\frac{T_b}{T_c}} \log P_c - 1 \quad \text{(S8)}$$

$$Z_{RA} = 0.29056 - 0.08775\omega \quad \text{(S9)}$$

$$V_s = \frac{RT_c}{P_c} \cdot Z_{RA}^{\left(1+\left(1-\frac{T}{T_c}\right)^{\frac{2}{7}}\right)} \quad \text{(S10)}$$

$$\rho = \frac{M}{V_s} \quad \text{(S11)}$$

where *R* represents the gas constant, 8.314 J/mol·K.

Standard enthalpy of combustion $\Delta_f H_{298} \mathrm{C}_j\mathrm{H}_{k(\mathrm{l})}$ can be calculated from Equations (S12) and (S13):

$$\Delta_f H_{298(\mathrm{l})} = \Delta_f H_{298(\mathrm{g})} - \Delta_{vap} H_{298(\mathrm{l})} \quad \text{(S12)}$$

$$\mathrm{C}_j\mathrm{H}_{k(\mathrm{l})} + \left(j + \frac{k}{4}\right) \mathrm{O}_{2(\mathrm{g})} \rightarrow j\mathrm{CO}_{2(\mathrm{g})} + \frac{k}{2} \mathrm{H}_2\mathrm{O}_{(\mathrm{g})} \quad \text{(S13)}$$

Net heat of combustion (NHOC):

$$NHOC = \Delta_f H_{298} \mathrm{C}_j\mathrm{H}_{k(\mathrm{l})}/M \quad \text{(S14)}$$

Volumetric net heat of combustion (V-NHOC):

$$V - NHOC = NHOC * \rho \quad \text{(S15)}$$

The specific impulse (I_{sp}/s) is calculated using Glushko's formula:

$$I_{sp} = \frac{(2 \times \eta NHOC)^{1/2}}{g} \quad \text{(S16)}$$

where η is the engine efficiency factor, which was taken equal to 0.556 as mainly defined for type RD-107 liquid-propellant rocket engines, and the acceleration of gravity (*g*) was 9.8 m/s^2.[2,11]

5. **XYZ coordinates of the designed fuel molecules**.

Table S3. XYZ coordinates of the designed fuel molecules.

Fuel Molecules	Atoms	X	Y	Z
ZD-1	C	0.09319500	-0.00056800	0.70063900
	C	-0.95819200	0.69847900	-0.13179700
	C	-2.09010700	-0.22997700	-0.20725000
	C	-1.05747500	-0.90811700	0.66500400
	C	0.19750100	-1.28130700	-0.13646700
	C	-0.12644500	-1.08736700	-1.60469300
	C	-1.21392900	-0.04225700	-1.45183500
	H	0.69050500	0.41664300	1.50084700
	H	-3.15160700	-0.12972300	-0.02192600
	H	0.76934300	-2.13398400	0.21511000
	H	0.70234500	-0.69530700	-2.20287300
	H	-0.46289600	-2.02827600	-2.05181400
	H	-1.55587500	0.55413800	-2.29151000
ZD-2	C	0.31645700	2.79050500	0.90825200
	C	-1.07102600	2.45195600	1.42070100
	C	-1.75328800	1.17460000	0.93102900
	C	-0.73315200	0.08056300	0.77851600
	C	0.46593000	-0.06820600	1.67075200
	C	0.62134000	0.96600000	2.77478100
	C	0.12676000	2.33964000	2.34317400
	C	-0.19945800	-0.56830900	-0.46121800

	C	0.66029900	0.42056800	0.26386300
	C	0.99393200	1.84492700	-0.08362000
	H	0.53138400	3.84377400	0.75604200
	H	-1.73932200	3.28971700	1.59469700
	H	-2.24544500	1.35746200	-0.03201200
	H	-2.54667200	0.88349600	1.63002100
	H	0.80492200	-1.07586400	1.92288400
	H	1.67550100	1.02864800	3.07138800
	H	0.07024600	0.63696200	3.66426800
	H	0.22010200	3.10842700	3.10380000
	H	-0.62852100	-0.23904000	-1.40539800
	H	0.06530000	-1.62667300	-0.44357700
	H	0.65538000	2.06527000	-1.10339300
	H	2.07785100	2.01188900	-0.07798500
ZD-3	C	-1.38292600	1.70857200	-1.88848400
	C	0.00902300	1.48080600	-1.29172700
	C	0.45664600	0.12511400	-0.72255200
	C	-0.70444200	-0.87236600	-0.76178000
	C	-1.79842300	-1.15807200	0.23477300
	C	-2.28133200	0.25500500	0.45442200
	C	-1.51647600	1.53680700	0.79713600
	C	-1.03385700	2.22590600	-0.48946400
	C	-2.08738300	-0.29624700	-0.93283300

	C	-2.39098100	0.54918100	-2.12304100
	H	-1.41866100	2.48102500	-2.65174200
	H	0.80756900	2.07385800	-1.72747500
	H	1.26675500	-0.27282000	-1.34023000
	H	0.88112500	0.26591700	0.27811000
	H	-0.52483300	-1.69994600	-1.45016300
	H	-2.15224400	-2.09835400	0.64789400
	H	-3.33741000	0.30979600	0.72408700
	H	-2.19862100	2.20701000	1.32786100
	H	-0.66722600	1.37501500	1.47087700
	H	-0.91091500	3.30165600	-0.40640400
	H	-2.18800400	0.05414400	-3.07853900
	H	-3.41634000	0.93360300	-2.14145600
ZD-4	C	-1.61885200	0.14437400	2.11571400
	C	-0.21807200	0.58122500	1.75267800
	C	0.31416200	0.01066100	0.45356600
	C	-0.49559700	-1.08044500	-0.14997500
	C	0.68779400	-1.47343000	0.64569600
	C	0.30017200	-1.83354500	2.08515100
	C	-0.40400300	-0.60035300	2.68516200
	C	-1.43587000	-1.23053900	-1.28845600
	C	-1.91523600	-1.37043600	0.15669700
	C	-2.58833300	-0.32937000	1.02703000

	H	-2.09860500	0.76174900	2.87113400
	H	0.16824700	1.53557300	2.09241000
	H	0.91070500	0.63986000	-0.19737500
	H	1.58683700	-1.88996800	0.20253300
	H	-0.30873600	-2.74275800	2.12965900
	H	1.20065000	-2.02099100	2.67947500
	H	-0.26178700	-0.46167700	3.75171700
	H	-1.68420900	-0.33124300	-1.84425100
	H	-1.42491500	-2.13774900	-1.88415800
	H	-2.11336200	-2.39964800	0.44741100
	H	-3.49373100	-0.73935400	1.48472400
	H	-2.90192900	0.54394800	0.44223400
ZD-5	C	-0.52053100	0.92914700	1.18137400
	C	0.42576400	0.85836900	-0.00957100
	C	0.98842700	-0.55447700	-0.32324400
	C	0.08935100	-1.68660500	0.08903600
	C	0.39253700	-2.61683200	1.23546500
	C	-0.71186400	-1.63185600	1.38757700
	C	-0.95839500	-0.29463500	1.99904300
	C	-1.92949300	0.40051700	1.06063900
	C	-2.25967100	-0.52824800	-0.12055000
	C	-1.41572900	-1.79337400	0.06890900
	H	-0.43534400	1.84397300	1.75977700

	H	-0.09580600	1.24558300	-0.89155000
	H	1.26724400	1.53503100	0.16155700
	H	1.23705800	-0.61861400	-1.38702600
	H	1.93324200	-0.68545100	0.21369300
	H	0.13684500	-3.67466500	1.16623400
	H	1.32263200	-2.42507700	1.76498200
	H	-1.05670200	-0.19552100	3.07322800
	H	-2.73450200	1.00909300	1.45756600
	H	-2.09572300	-0.04175100	-1.08854900
	H	-3.31707100	-0.80320500	-0.08307500
	H	-1.86784000	-2.74426400	-0.21832100
ZD-6	C	2.67671600	-0.74881800	1.33857400
	C	2.42585800	0.76114700	1.40170100
	C	0.96424900	1.21592100	1.65542800
	C	0.37837600	0.30944200	2.72614500
	C	-0.78709900	-0.62240700	2.64037600
	C	0.60473000	-1.18930000	2.62095600
	C	1.90454000	-1.78418500	2.15760500
	C	1.80956600	-1.75073500	0.61216600
	C	0.37301100	-1.36366400	0.16707800
	C	-0.42075400	-1.47067100	1.48592800
	H	3.73903100	-0.96802300	1.26071200
	H	3.05533400	1.15816400	2.20817100

	H	2.80060100	1.21425200	0.47726800
	H	0.97231700	2.25544000	1.99907900
	H	0.37897400	1.20694600	0.73749100
	H	0.57278800	0.67694400	3.73633900
	H	-1.65235400	-0.72004800	3.29656900
	H	2.44089800	-2.57319900	2.67375100
	H	2.32665700	-2.50697500	0.02928400
	H	-0.00628300	-2.10283200	-0.54479000
	H	0.33956100	-0.40144300	-0.34092900
	H	-0.83510600	-2.47771700	1.57872100
ZD-7	C	1.37084700	0.28498600	1.65672000
	C	1.67057100	1.62053600	0.93919600
	C	0.45115200	2.26014300	0.25008700
	C	-0.79115300	2.02301800	1.05009500
	C	-1.97098700	2.10094300	1.96676800
	C	-0.66063100	1.43929500	2.40596400
	C	0.03037100	0.11041000	2.40438300
	C	0.30740500	-0.63135700	1.10912400
	C	-0.36965600	-0.23658900	-0.18543100
	C	-0.60546500	1.25723800	-0.21100800
	H	2.24721400	-0.17790400	2.09916000
	H	2.44891600	1.44128100	0.18738900
	H	2.10066700	2.32753600	1.66066600

	H	0.63916000	3.14082400	-0.35924400
	H	-2.22818000	3.00980400	2.50270900
	H	-2.82480400	1.50840600	1.65349600
	H	-0.14096800	2.12538100	3.07926500
	H	0.04859700	-0.48106700	3.31529700
	H	0.48695200	-1.69560600	1.22690500
	H	0.24597800	-0.55421100	-1.03751300
	H	-1.33022000	-0.75832900	-0.28408700
	H	-1.22786000	1.61600100	-1.02836400
ZD-8	C	0.18445000	-1.02749400	-2.09123700
	C	1.13762100	-0.96026800	-0.91447900
	C	0.78596200	-0.04764000	0.23974000
	C	-0.59399700	0.50803300	0.39973000
	C	-1.79287400	-0.41261700	0.45159700
	C	-1.54275900	0.44111800	-0.77158400
	C	-1.06686200	-0.17831700	-2.04770200
	C	0.16712000	0.33357500	-2.76603700
	C	1.14550800	1.24959200	-2.02344500
	C	1.71687600	0.44287700	-0.85275200
	H	0.16797900	-1.94383900	-2.66908200
	H	1.74941900	-1.83230200	-0.71668100
	H	1.28062100	-0.28791200	1.17748500
	H	-0.65054800	1.40402300	1.01155600

	H	-1.60567600	-1.47655600	0.36074700
	H	-2.61714500	-0.14926000	1.10420900
	H	-2.20187400	1.29461000	-0.90366700
	H	-1.86546900	-0.50980200	-2.70658700
	H	0.11952000	0.41571900	-3.84655600
	H	0.67653400	2.18420200	-1.69697000
	H	1.96139500	1.52116800	-2.69982700
	H	2.75722300	0.60175300	-0.59012500
ZD-9	C	0.64533100	-0.17428500	0.62260300
	C	1.44426500	-0.68237900	-0.60642000
	C	0.92905100	-0.26648300	-2.03796600
	C	-0.51801800	-0.02133700	-1.79226700
	C	-1.90967000	0.46323300	-1.76705700
	C	-0.87965500	1.11869700	-0.87624300
	C	-0.52964400	0.81587700	0.54952500
	C	-0.77604200	-0.60119300	0.98308600
	C	-1.64828600	-1.45918700	0.08208900
	C	-1.60963800	-0.96236800	-1.35828600
	H	1.28138500	-0.06692000	1.49612800
	H	1.46041600	-1.77680100	-0.56224000
	H	2.48369400	-0.35970600	-0.49104500
	H	1.42921500	0.64439900	-2.38022100
	H	1.14220700	-1.05986600	-2.75944300

	H	-2.62883500	0.87681900	-2.46489900
	H	-0.51166700	2.08213400	-1.23186300
	H	-0.58546300	1.61441500	1.28296600
	H	-0.93595900	-0.75710400	2.04557600
	H	-2.67392700	-1.46514500	0.47167200
	H	-1.30502800	-2.50123300	0.10364900
	H	-1.87859600	-1.71051400	-2.10402600
ZD-10	C	-2.31339200	-0.03827400	1.89839000
	C	-1.58704500	-0.70402600	0.82894400
	C	-1.36205100	-0.77346200	-0.65527600
	C	-0.14278600	0.10461500	-1.03166100
	C	0.98107500	0.00072300	0.01929000
	C	0.54409100	0.54000800	1.38764500
	C	-0.89202300	0.33465100	1.72081900
	C	0.10876400	-0.34685600	2.59360200
	C	-0.48502700	-1.76239100	2.71248200
	C	-1.85754500	-1.55564400	2.00529400
	H	-3.27340600	0.44437900	2.01829100
	H	-2.24398100	-0.47213600	-1.22940900
	H	-1.14957700	-1.81413800	-0.92394000
	H	-0.45331200	1.15223600	-1.10976900
	H	0.23192100	-0.18910200	-2.01680100
	H	1.86000500	0.55276800	-0.32447800

	H	1.28940200	-1.04655000	0.11868400
	H	1.04769900	1.45183400	1.70599800
	H	0.30690500	0.14792900	3.54206800
	H	0.06590700	-2.55759800	2.19586500
	H	-0.55139500	-2.04475500	3.76797200
	H	-2.61953000	-2.33363500	1.96959200
ZD-11	C	0.23767900	-0.80277900	-0.81555300
	C	1.06329000	-1.24825600	-2.01577100
	C	0.37864700	-1.66152800	-3.29406300
	C	1.06758000	-2.69686100	-2.43367500
	C	0.99069100	-1.06997100	0.50956800
	C	1.95801900	0.05662700	0.85604400
	C	2.32325300	1.09423100	-0.17361000
	C	0.93307800	1.61253000	-0.42940700
	C	1.44777400	1.46412600	0.97492300
	C	-0.17751400	0.69052000	-0.94128100
	C	-1.48842400	0.99567000	-0.23453300
	C	-2.36065600	2.13585500	-0.69940500
	C	-2.80848900	0.70683000	-0.90330200
	H	-0.67909000	-1.40399500	-0.81849000
	H	2.00536700	-0.72354400	-2.13786400
	H	-0.70616900	-1.66580100	-3.30796400
	H	0.83336900	-1.39290200	-4.24040900

	H	0.43839100	-3.38458100	-1.87864600
	H	1.99168400	-3.13359400	-2.79390700
	H	0.28024900	-1.19105500	1.33538600
	H	1.53244300	-2.01744100	0.42258100
	H	2.75076700	-0.20920800	1.55297500
	H	3.28427600	1.49559400	-0.45624600
	H	0.87284400	2.63728900	-0.79120200
	H	1.61234000	2.20593900	1.74106500
	H	-0.32277900	0.89651200	-2.00928300
	H	-1.45931100	0.84167800	0.84072700
	H	-2.87336900	2.73555200	0.04337600
	H	-2.04237700	2.69067000	-1.57549300
	H	-2.78776800	0.31399200	-1.91422400
	H	-3.62714700	0.33396500	-0.29896900
ZD-12	C	-1.71953000	-0.01347900	1.31410200
	C	-2.27845400	-0.95665200	0.26159700
	C	-3.39110500	-1.91592200	0.60699500
	C	-3.65831900	-0.74234800	-0.30683000
	C	-1.12312700	1.28033100	0.72065600
	C	0.31413000	1.10763600	0.19237500
	C	0.96937600	2.45450800	-0.05599700
	C	2.08049300	2.59693600	-1.06730800
	C	0.72403600	3.20369900	-1.34135700

	C	1.12433200	0.26320700	1.18093100
	C	0.59987300	0.00109900	2.57123600
	C	-0.66506100	-0.71508700	2.17421000
	C	0.65267000	-1.10618900	1.57045500
	H	-2.55119500	0.27738300	1.96966900
	H	-1.53481200	-1.33824600	-0.43253400
	H	-3.37306400	-2.91020300	0.17656100
	H	-3.80143500	-1.87389900	1.61028700
	H	-4.24543200	0.07957400	0.08860900
	H	-3.82313400	-0.93846900	-1.35981200
	H	-1.77438000	1.67751200	-0.06323600
	H	-1.09007700	2.03050600	1.51861200
	H	0.28937200	0.56871700	-0.76472200
	H	1.06466300	3.06904500	0.83627700
	H	2.36186700	1.71758100	-1.63651600
	H	2.90505600	3.26318800	-0.84255800
	H	0.10206400	2.72629500	-2.09073000
	H	0.62586900	4.28215800	-1.30450800
	H	2.20374300	0.37860800	1.09836100
	H	1.09157800	0.07130000	3.52909300
	H	-1.06218000	-1.41557600	2.90641900
	H	1.19313800	-2.03869800	1.62323400
ZD-13	C	-0.27340400	0.88465200	-0.88175600

	C	-1.22477200	1.20266600	0.24342500
	C	-2.31478600	2.20568100	-0.03224000
	C	-2.55112400	3.38160200	0.88729000
	C	-2.04896900	3.60676200	-0.51916700
	C	-1.66107100	0.05553400	1.16218900
	C	-0.45514800	-0.64874600	1.80130800
	C	0.68928200	-0.89404200	0.79772700
	C	0.48230800	-2.13016300	-0.06159000
	C	1.66557800	-2.86578100	-0.64125700
	C	0.84536500	-3.49742900	0.45979100
	C	0.86993400	0.35346700	-0.07077500
	C	0.21568300	1.65997300	0.30048300
	H	-0.31207200	1.19669100	-1.91443400
	H	-3.21711900	1.74376700	-0.42532200
	H	-3.57174900	3.65971200	1.12260300
	H	-1.84751400	3.52925200	1.69837200
	H	-2.73197700	4.02894800	-1.24676200
	H	-1.02077600	3.92356600	-0.64458000
	H	-2.26836600	-0.64933600	0.58166700
	H	-2.30975300	0.44934000	1.95202500
	H	-0.07072100	-0.00167900	2.59640600
	H	-0.75759100	-1.58862000	2.27258500
	H	1.61274700	-1.04302900	1.37282400

	H	-0.39536900	-2.08505900	-0.70029700
	H	1.58525200	-3.27357400	-1.64197900
	H	2.65748200	-2.50856500	-0.38579200
	H	0.20701600	-4.33617800	0.20797400
	H	1.29139800	-3.55988300	1.44661200
	H	1.84532200	0.44271600	-0.54536800
	H	0.63856800	2.65238700	0.32744100
ZD-14	C	-1.70536300	0.96118200	0.04636700
	C	-1.73176300	-0.47041800	0.57345900
	C	-1.43405800	-1.46415800	-0.56080700
	C	-0.27461100	-0.99854800	-1.46768500
	C	1.09608400	-1.31119800	-0.89017100
	C	2.28942200	-1.48345400	-1.79885000
	C	1.71194800	-2.67536900	-1.07119600
	C	-0.42570400	0.49837900	-1.76217800
	C	-1.71035300	1.21917200	-1.44111500
	C	-0.48229200	1.50312300	-0.64244200
	C	0.26451900	2.75948000	-0.39290100
	C	1.37213300	3.22177200	-1.31092800
	C	1.70190600	2.74344200	0.08111800
	H	-2.24126300	1.69208100	0.65098800
	H	-2.71395600	-0.69464500	1.00369200
	H	-1.00539100	-0.57069400	1.38842800

	H	-1.22940900	-2.46303100	-0.16465800
	H	-2.33245100	-1.54326300	-1.18141300
	H	-0.35999200	-1.53838400	-2.42021800
	H	1.29957700	-0.82554700	0.06028800
	H	3.25037800	-1.10002800	-1.47661800
	H	2.12260700	-1.41994400	-2.86879200
	H	2.28050900	-3.10173800	-0.25309400
	H	1.16152000	-3.40403300	-1.65655100
	H	0.09229900	0.84163000	-2.65585500
	H	-2.30654800	1.88908900	-2.04214500
	H	-0.35114200	3.55000500	0.02984400
	H	1.44535300	4.28147600	-1.52467200
	H	1.64897400	2.58034100	-2.13855300
	H	2.18378200	1.77755000	0.17444000
	H	2.00649200	3.47419900	0.82115200
ZD-15	C	-0.24176900	-0.56807100	1.70989200
	C	0.80705900	-1.62796800	2.02419700
	C	1.17415800	-2.39213500	0.74519300
	C	1.37152100	-1.45353600	-0.46364400
	C	2.73553900	-0.77751600	-0.45421800
	C	3.47014800	-0.46158200	-1.73614000
	C	3.97524900	-1.55345400	-0.82454500
	C	0.22646100	-0.41682200	-0.52113500

	C	0.01538200	0.19354300	-1.88453600
	C	-1.27994500	0.83382700	-2.31378600
	C	-0.85573600	-0.48332300	-2.91827000
	C	0.02594500	0.48206300	0.67345800
	C	-0.96763200	-0.59854000	0.38934300
	H	-0.83906100	-0.23327200	2.55599300
	H	1.68798300	-1.15165600	2.46952000
	H	0.41873500	-2.32858700	2.77121000
	H	2.06670000	-3.00611400	0.89583500
	H	0.35021000	-3.07417700	0.51125900
	H	1.30880800	-2.06568600	-1.37262300
	H	2.86128300	-0.04922700	0.34208900
	H	3.01161700	-0.76510400	-2.67061400
	H	4.03449700	0.46181500	-1.79680900
	H	4.88531900	-1.37747100	-0.26314400
	H	3.84854900	-2.57919700	-1.15393900
	H	0.91042100	0.66431500	-2.27757100
	H	-2.11632300	0.82835500	-1.62585000
	H	-1.22346600	1.72320300	-2.93031000
	H	-1.39785200	-1.36993200	-2.61000900
	H	-0.52387500	-0.49685000	-3.94991300
	H	-0.13924400	1.54855000	0.70264600
	H	-2.02330800	-0.50795700	0.18611600

ZD-16	C	-0.89491700	-0.76617900	-0.92288300
	C	-2.29009000	-1.04569900	-1.44454500
	C	-2.88900600	-2.42679400	-1.34179000
	C	-2.49613500	-1.87152500	-2.69052600
	C	-0.87479300	-0.53495300	0.59851800
	C	0.48575700	-0.02488700	1.09170100
	C	0.71704300	-0.25034700	2.56351400
	C	2.08461900	-0.25428600	3.19644900
	C	1.30717000	-1.54357400	3.07683100
	C	1.02776700	1.24598100	0.48991600
	C	1.06909200	0.81425900	-0.94316200
	C	1.65716800	-0.06258400	0.13585900
	C	-0.21316500	0.40029200	-1.65810900
	H	-0.28041700	-1.65669500	-1.11173500
	H	-2.98768600	-0.22229400	-1.30887800
	H	-2.27104200	-3.21362600	-0.92310500
	H	-3.94350300	-2.51992800	-1.11045800
	H	-3.28277500	-1.58585100	-3.37883300
	H	-1.61709700	-2.28936500	-3.16907500
	H	-1.64851000	0.18990400	0.88359500
	H	-1.12078100	-1.47367800	1.10623000
	H	-0.07584500	0.16694100	3.17933500
	H	2.94962300	-0.12446900	2.55752800

	H	2.19064600	0.19213700	4.17807100
	H	0.89239600	-1.98257200	3.97661400
	H	1.64532000	-2.26372000	2.34078900
	H	1.47713300	2.09625500	0.98027800
	H	1.79894300	1.33624200	-1.55916000
	H	2.67677500	-0.38536000	0.27933300
	H	-0.88947200	1.26235100	-1.71794600
	H	0.00641200	0.10028500	-2.68826900
ZD-17	C	-0.99153200	0.91423200	0.20339000
	C	-0.10084600	1.46757500	-0.90145200
	C	0.55238300	2.81722600	-0.74371800
	C	-0.57133000	2.63038400	-1.73819900
	C	-2.01411100	-0.09986100	-0.36145300
	C	-1.42513500	-1.50358300	-0.47391700
	C	-0.86688900	-2.17765500	0.74720500
	C	0.21186000	-1.17467300	1.04553800
	C	0.06011900	-1.75473800	-0.34038500
	C	0.98285200	-2.52003800	-1.21237800
	C	2.36822700	-2.91299400	-0.75431300
	C	2.20257200	-1.87799600	-1.83936800
	C	-0.15953200	0.27626100	1.34153700
	H	-1.54321700	1.76567300	0.61942100
	H	0.48373200	0.72164200	-1.42955200

	H	1.55753700	2.95917400	-1.12331500
	H	0.33508700	3.38134500	0.15712700
	H	-0.33100200	2.64345700	-2.79483900
	H	-1.53280700	3.07069900	-1.49613600
	H	-2.89990100	-0.14251000	0.28346000
	H	-2.35472400	0.24712000	-1.34248300
	H	-1.93509800	-2.16334000	-1.17499900
	H	-1.04038500	-3.16248700	1.15437300
	H	1.05072500	-1.56141100	1.62159600
	H	0.47556300	-3.25112200	-1.83698200
	H	2.70127400	-2.57057500	0.21783800
	H	2.73331700	-3.89559500	-1.02852400
	H	2.41390900	-0.84763200	-1.57921800
	H	2.46021800	-2.14797700	-2.85675300
	H	0.74537100	0.87257900	1.49858000
	H	-0.72345700	0.30624100	2.28144800
ZD-18	C	-0.29977300	1.77714800	0.75504800
	C	-0.43993700	2.17574200	-0.69428200
	C	0.30353200	0.93413500	-0.33175300
	C	1.69370100	0.47026900	-0.55888100
	C	2.81133500	0.82641500	0.39463900
	C	2.30849600	-0.59366000	0.32682600
	C	-0.91835800	0.83892600	-1.22304100

	C	-0.69668300	0.49073900	-2.67315400
	C	-1.40469300	1.25491000	-3.76856700
	C	0.09511300	1.34807200	-3.62884500
	C	-2.20808400	0.23647400	-0.64574700
	C	-2.67770600	0.99548200	0.60217100
	C	-1.51041500	1.29660400	1.55031300
	H	0.42000000	2.36812500	1.31879900
	H	-0.10399300	3.06801100	-1.20064000
	H	1.97376300	0.41721900	-1.60583500
	H	3.78396200	1.05093300	-0.02691600
	H	2.56703400	1.40312700	1.27827200
	H	1.71382700	-0.95345600	1.15827200
	H	2.93874100	-1.34474300	-0.13480900
	H	-0.61846000	-0.58052200	-2.84322200
	H	-1.98799700	2.11998200	-3.47486300
	H	-1.81235100	0.70270300	-4.60712900
	H	0.51143300	2.27935400	-3.26533800
	H	0.71121700	0.84848300	-4.36749700
	H	-2.99503200	0.26214600	-1.40707100
	H	-2.02998600	-0.82104400	-0.41296000
	H	-3.47252800	0.44715200	1.11658600
	H	-3.10848200	1.94926000	0.27862500
	H	-1.23672800	0.40451600	2.12690400

	H	-1.80929300	2.06064000	2.27613500
ZD-19	C	-0.24082800	0.20159800	-0.21079700
	C	0.48467500	-1.07953800	0.09620800
	C	0.93487500	-1.29281500	1.51902200
	C	1.82362400	-0.33176900	2.26510200
	C	2.35856400	-1.68062600	1.84565100
	C	0.02097200	-2.36532400	-0.59796700
	C	0.05733700	-2.21801900	-2.12341100
	C	-0.50772400	-0.86681400	-2.57612800
	C	0.00663300	0.27540200	-1.69242800
	C	-0.09700600	1.63189800	-2.34184800
	C	0.70870200	2.82830800	-1.90646900
	C	1.00149800	2.16577500	-3.23192500
	C	1.14700600	-0.00372700	-0.73725800
	H	-0.58294700	0.97207200	0.46429500
	H	0.17723700	-1.78438200	2.12441600
	H	1.61998400	-0.16446000	3.31613200
	H	2.19218800	0.54313000	1.74373800
	H	3.06914300	-1.70341200	1.02753000
	H	2.53099900	-2.42890600	2.61036300
	H	-0.99243300	-2.60565600	-0.25289300
	H	0.66519800	-3.19676700	-0.29271900
	H	1.10191000	-2.27707500	-2.44732700

	H	-0.47112400	-3.04444400	-2.60821100
	H	-1.60439300	-0.87114800	-2.54259100
	H	-0.22323700	-0.67862900	-3.61687500
	H	-1.10652400	1.85424500	-2.67888100
	H	1.42997300	2.70351500	-1.10815500
	H	0.22245400	3.79663000	-1.91284400
	H	1.91056700	1.58039900	-3.30738100
	H	0.72635200	2.68609400	-4.14187300
	H	2.03476700	0.57555100	-0.53570700
ZD-20	C	-0.95943900	0.23682400	-0.74810000
	C	-0.52533500	-0.46609500	-2.03312300
	C	0.83362900	0.07126500	-2.50735300
	C	1.81965800	0.23081600	-1.33977500
	C	1.12193100	0.86051900	-0.13490900
	C	-0.08779300	0.20723600	0.47893300
	C	-0.41408400	-0.19265300	1.86809300
	C	0.20247000	-1.42592200	2.49400500
	C	0.59228900	-0.05546800	2.98783000
	C	-0.24974500	1.48650500	-0.27104900
	C	-0.73314300	2.83171000	0.11920200
	C	-2.18876200	3.21731700	-0.01561600
	C	-1.15995300	3.84358100	-0.92292100
	H	-2.02763300	0.19995200	-0.53633000

	H	-1.27469900	-0.31551300	-2.81811200
	H	-0.47344500	-1.54678400	-1.85232000
	H	1.25424100	-0.56462300	-3.29187500
	H	0.67289900	1.05920800	-2.95276500
	H	2.66299200	0.85531400	-1.65385800
	H	2.23888700	-0.74116400	-1.05161300
	H	1.77977600	1.36338500	0.57321100
	H	-1.44822400	-0.00296100	2.14498200
	H	0.92934900	-1.97192100	1.90433800
	H	-0.42061300	-2.04572800	3.12807000
	H	0.23216900	0.26559500	3.95802600
	H	1.58371100	0.30372900	2.74018300
	H	-0.21882100	3.24732000	0.98213100
	H	-2.62768400	3.82653300	0.76559000
	H	-2.86784500	2.49439500	-0.45089600
	H	-0.89742900	4.88331900	-0.76656000
	H	-1.14509800	3.52554300	-1.95875000

6. **Calculations of the electronic and thermal corrections to the energies, enthalpies, and free energies of the designed fuel molecules.**

Table S4. Electronic and thermal corrections to the energies, enthalpies, and free energies of the designed fuel molecules.

Fuel Molecules	Electronic Energy	Thermal Correction to	Thermal Correction to Enthalpy	Thermal Correction to Gibbs Free Energy

	(Hartree)	Energy (Hartree)	(Hartree)	(Hartree)
ZD-1	-270.158560	0.106996	0.107940	0.074611
ZD-2	-388.279904	0.200167	0.201111	0.162415
ZD-3	-388.220566	0.199223	0.200167	0.161678
ZD-4	-388.265652	0.199301	0.200246	0.161420
ZD-5	-388.263179	0.199806	0.200750	0.161426
ZD-6	-388.180690	0.198284	0.199228	0.160192
ZD-7	-388.247057	0.199566	0.200511	0.161847
ZD-8	-388.291457	0.199958	0.200902	0.162347
ZD-9	-388.239913	0.199503	0.200447	0.161386
ZD-10	-388.189119	0.197812	0.198756	0.158764
ZD-11	-506.205797	0.287012	0.287956	0.237485
ZD-12	-506.208617	0.286955	0.287899	0.237177
ZD-13	-506.209092	0.286826	0.287770	0.237110
ZD-14	-506.212015	0.287217	0.288161	0.236907
ZD-15	-506.207676	0.286903	0.287847	0.237497
ZD-16	-506.210140	0.286686	0.287630	0.236945
ZD-17	-506.209766	0.287185	0.288130	0.237048
ZD-18	-506.212395	0.287080	0.288024	0.236909
ZD-19	-506.210113	0.286585	0.287530	0.236901
ZD-20	-506.216090	0.287464	0.288408	0.236371

7. **The differences in strain energy values between the PM7 and DFT methods for designing the fuel molecules.**

Table S5. The differences in strain energy values between the PM7 and DFT methods for designing the fuel molecules

Fuel Molecules	Strain Energy Using PM7 (kJ/mol)	Strain Energy Using DFT (kJ/mol)
ZD-1	1031.62052	767.7828515
ZD-2	484.64156	433.099876
ZD-3	564.20751	551.4911005
ZD-4	436.0362	433.3231005
ZD-5	526.98872	476.063876
ZD-6	659.4279	653.7181005
ZD-7	479.15834	482.8421005
ZD-8	369.9005	332.375325
ZD-9	532.0773	501.4311005
ZD-10	686.28403	665.272876
ZD-11	550.72957	525.1205495
ZD-12	550.51831	517.5675495
ZD-13	550.85055	550.903325
ZD-14	543.4238	544.255325
ZD-15	549.48338	554.823325
ZD-16	548.15091	547.784325
ZD-17	544.06125	550.079325
ZD-18	543.78441	577.8201005

ZD-19	548.8538	582.5141005
ZD-20	539.10549	569.1301005

Reference

1. Landrum, G. Rdkit: Open-Source Cheminformatics. http://www.rdkit.org (accessed 09.1).
2. Stewart, J. J., Optimization of Parameters for Semiempirical Methods Vi: More Modifications to the NDDO Approximations and Re-Optimization of Parameters. *J. Mol. Model.* **2013**, *19*, 1-32.
3. Tan, B.; Long, X.; Li, J., The Cage Strain Energies of High-Energy Compounds. *Comput. Theor. Chem.* **2012**, *993*, 66-72.
4. Tan, B.; Huang, M.; Long, X.; Li, J.; Yuan, X.; Xu, R., From Planes to Cluster: The Design of Polynitrogen Molecules. *Int. J. Quantum Chem.* **2015**, *115*, 84-89.
5. Coley, C. W.; Rogers, L.; Green, W. H.; Jensen, K. F., Scscore: Synthetic Complexity Learned from a Reaction Corpus. *J. Chem. Inf. Model.* **2018**, *58*, 252-261.
6. Catoire, L.; Naudet, V., A Unique Equation to Estimate Flash Points of Selected Pure Liquids Application to the Correction of Probably Erroneous Flash Point Values. *J. Phys. Chem. Ref. Data* **2004**, *33*, 1083-1111.
7. Osmont, A.; Gökalp, I.; Catoire, L., Evaluating Missile Fuels. *Propellants, Explos., Pyrotech.* **2006**, *31*, 343-354.
8. Savos'kin, M. V. et al., New Approaches to the Development of High-Performance Hydrocarbon Propellants. *Russ. J. Appl. Chem.* **2007**, *80*, 31-37.
9. Saldana, D. A.; Starck, L.; Mougin, P.; Rousseau, B.; Pidol, L.; Jeuland, N.; Creton, B., Flash Point and Cetane Number Predictions for Fuel Compounds Using Quantitative Structure Property Relationship (QSPR) Methods. *Energy Fuels* **2011**, *25*, 3900-3908.
10. Lee, C.; Yang, W.; Parr, R. G., Development of the Colle-Salvetti Correlation-Energy Formula into a Functional of the Electron Density. *Phys. Rev. B: Condens. Matter Mater. Phys.* **1988**, *37*, 785-789.
11. Li, G.; Hu, Z.; Hou, F.; Li, X.; Wang, L.; Zhang, X., Machine Learning Enabled High-Throughput Screening of Hydrocarbon Molecules for the Design of Next Generation Fuels. *Fuel* **2020**, *265*, 116968.
